# Nature and Use of Carbolic Acid

**Published:** 1869-02

**Authors:** 


### THE NATURE AND USES OF CARBOLIC ACID.

---

[From the Half-Yearly Compendium of Medical Science, July, 1868.]

AT the April meeting of the New York Medical Journal Association (*N. Y. Medical Gazette*, April 18), E. R. Squibb, M. D. made some remarks on the "so-called carbolic acid," from which we take the following:

"The carbolic acid," was originally discovered many years ago by Runge, as a product of coal-tar distillation. It was further investigated by Laurent. Runge gave it the name of carbolic acid, while Laurent called it the hydrate of phenyl. Really it is not an acid but an alcohol: its peculiarity of combining with bases is not enough to constitute it an acid. The alcohols are now just as distinct a class as are acids or alkalies. An alcohol is a compound of carbon, hydrogen, and oxygen, formed on the type of water, in which one half of the hydrogen of the water is replaced by a radical; these radicals being organic compounds; *e.g.*, ethyl, methyl, etc. These radicals now run through chemistry as do the acids and alkalies. This compound then that we know as carbolic acid is really phenyl alcohol, formed on the radical phenyl, and is found in coal-tar.

"In the distilleries, the coal-tar is pumped warm into large retorts, and the volatile products distilled off. These products are collected in three groups:

"*a.* The 'light oils.' To these belong the benzine, or benzole, now employed in arts very extensively as a solvent, and as a substitute for alcohol, in processes where the latter would be too costly. In Europe it is used, in addition, for the manufacture of analine dyes, which are as yet produced there at less expense. These light oils distil off at temperatures of about 70° to 180° C.

"*b.* The 'dead oils' or 'heavy oils.'

"*c.* The residue, which is the artificial asphalt used for roofing and similar purposes. The first and third groups are the vaulable ones and the objects of the distillation. The second group, the 'dead oils,' are considered a waste, and are very cheap, and generally lie about the distillery yards in great quantity. It is from this second group that the tar alcohols are commonly obtained.

"If an alkali be mixed with the dead oil, a separation into two strata takes place, one of which is a compound of tar alcohols with the alkali. The alkali is then separated by treating with an acid, and the product is an impure carbolic acid.

"The purification of this consists in separating the phenylic alcohol from tar and oils, and from the cresylic. This is not necessary if a disenfectant or antizymotic is required, as the cresyl alcohol is probably better for these purposes than the pure phenyl alcohol. The cresylic differs from the phenylic in boiling at a higher point, being less soluble in water, and being as yet uncrystallizable. Any dehydrating substance, such as chloride of calcium, appears to change it into phenyl alchhol. Nearly, if not quite all the experiments that have been made with the carbolic acid, for disenfecting purposes on the large scale, have really been made with a mixture of the two. A mixture of them with some other alcohols is sold under the name of coal-tar creasote, which is, chemically, quite different from wood creasote, but medicinally, very similar.

"The carbolic acid when nearly pure, is a crystalline, moist solid, often of a pink or red color, having in the bottom of the bottle a certain quantity of liquid at ordinary temperature. This liquid, if the specimen be a good one, should not exceed one-fourth of the whole. From two to five per cent, of water will liquefy the whole of the crystals.

"The unseparated mixture of the two or more alcohols varies from a sherry wine color to almost black, and frequently is quite opaque, unless in a very thin layer. This mixture as a disinfectant, is as good as the pure alcohol, if not better. The test for the mixture is its solubility in water: a small quantity shaken up with two or three hundred parts of water, has the soluble tar alcohols dissolved out, and the proportion of residue left is the measure of its impurity. It is often adulterated with an alkali to increase its solubility.

"In ordering a saturated solution for use, it should be expressly stated whether the impure or the pure carbolic acid is desired, as the former is soluble to the amount of about two per cent., and the latter about six and a half per cent. The latter, if applied to the delicate skin of women or children acts as a caustic irritant.

"A standard solution may be made be dissolving two pints of the impure acid in a barrel (forty gallons) of water. This is about the strength of the preparations of the acid sold as disenfectants, and quite as effectual, at far less cost. The impure mixture contains from seventy to ninety per cent. of the alcohols. For internal use, the crystallized acid is preferred as being more elegant, though as a remedy for vomiting and sarcinæ ventriculi, it is not so effectual as when the mixture of the group of alcohols is given. Mr. William Crookes, in his report to Parliament on the cattle plague, expresses the belief that cresyl alcohol is more powerful as an antizmotic. than the phenyl alcohol, and Dr. Angus Smith arrives at the same conclusion.

"The carbolic acid was first brought to the notice of the profession as a therapeutic agent by Mr. F. Crace Calvert, of Manchester. He first took the matter up in a business way and rendered the acid obtainable.

"The prominent characteristic of the group is that it is deadly poison to the lower orders of animal and vegetable life. No cryptogams can live near it. The following experiment illustrates its peculiar character and indicates its uses: A large bottle, containing a little syrup, was allowed to become well covered with a growth of mould. A narrow slip of paper, the end of which was moistened with a solution of the impure carbolic acid, or coal-tar creasote, was suspended in the air space of the bottle above the cryptogams, and solutions of varying strength were used on the end of the paper. Until a proportion of one part in eight hundred was reached no marked effect on the mould was noticed, but in the proportion of one part in five hundred of water the whole growth was arrested in a single night, probably by less than a single drop of the solution suspended above it, and both the plants and spores were so killed that the moulding did not recommence. The same effect is produced upon animalcula and fish in water.

"This points to its use in all forms of parasitic skin disease. In the cryptogamous forms of pityriasis, and psoriasis tinea favosa, and also in scabies, it is found useful. Dr. Gray, of the State Lunatic Asylum, writes he has used it largely, and esteems it highly. He uses a solution of about the standard strength, and remarks that if it had no other use it would be invaluable in destroying vermin. For this purpose he uses it in the form of baths containing a small amount of the standard solution.

"It also is useful in cleaning away the tartar which gathers on the teeth, and which is due to animalcula.

"It has the power, too, of arresting all forms of cryptogamous fermentations. There are two kinds of fermentation, the one purely chemical and the other cryptogamous; an example of the latter is found in the action of yeast; of the former, the change of starch into dextrine. That form of cryptogamous fermentation known as yeasty vomiting is speedily arrested by the carbolic acid, which destroys the sarcinæ ventriculi. It is also fatal to animal life of as high a grade as the ascarides (oxyrues vermiculares). For their destruction a weak solution can be injected into the rectum. In some instances, however, the remedy will fail.

"Some of the applications of carbolic acid are really but revivals of old methods of practice; thus creasote has a very deservedly high reputation as an application to burns, and as carbolic acid is medicinally at least identical with creasote, it is but changing the name of the agent.

"In my own person I have often experienced its benefits, and once tried the following experiment: Accidentally scalded by a jet of steam, a solution of carbolic acid, which is kept constantly at hand for such purposes, was at once applied on a piece of lint, before the pain became severe. In two or three minutes the pain was gone. In an hour the dressing was removed and the pain then returned. The dressing was renewed and the pain again ceased, but to return again when the dressing was removed. This anodyne effect has not been explained as yet, and does not appertain to its peculiar character as an antizymotic. A boy employed in my factory broke a bottle of compound spirits of ether, saturating the front of his pantaloons; this then took fire from a lamp which he was using, and he was quite severely burned over the lower part of the abdomen, the genitals, and the upper part of the thighs. In my absence he was taken to his home and dressed with cotton and oil. In a few hours I obtained permission of his medical attendant to apply the carbolic acid, which was attended with speedy relief of his pain, and he had a quick recovery. This application of the carbolic acid is one of its most valuable uses.

"Again the carbolic acid arrests the process of suppuration' which is perhaps, a form of fermentation; but its *modus operandi* we are ignorant of. Sulphurous acid acts by deoxidation. Carbolic acid acts by catalysis apparently, which is only another way of expressing ignorance.

"It will not always arrest suppuration, but generally does so, particularly if it be chronic. Thus in chronic cystitis, which continues, as it were from habit, after all inflammation has ceased, the secretion of pus will frequently be arrested by the use of injections of a solution of carbolic acid. The particular indication cannot always be pointed out, but it succeeds in some cases. In using the injections in this disease, it is better to begin with one-quarter of the strength of the saturated solution of the impure or one-twelfth of pure acid; but, as no rule can be definitely laid down, the strength may have to be considerably increased, and the full strength should be used before this plan of treatment is abandoned. There may be some pain experienced after the use of a weak injection; this pain is, generally, only momentary, and is not increased by increasing the strength of the injection within reasonable limits. Its use as an injection in gonorrhœa often arrests the discharge, sometimes with wonderful quickness. Dr. Hutchison has employed it in a number of cases, but has not yet collected data enough to draw any very positive conclusions. If, as Dr. Salisbury maintains there is a specific form of plant-growth producing each form of venereal disease, we should expect carbolic acid to be very effectual. It was formerly used as a local application to syphilitic ulcers, and internally for gonorrhœa; but the use referred to above, of injections, is of recent introduction. How it would act in deeper-seated inflammation of the urethra I do not know.

"As a local application to aphthæ, and to the exudation of diphtheria, it has been much vaunted, and is very likely of value.

"The Egyptian processes of embalming bodies by burning spices, I think, owed its efficacy to the creasote produced in this combustion. Some French writer states that a single gramme of carbolic acid is sufficient to preserve a human body, but he does not state for how long a time. The preservative power of carbolic acid is rather antiseptic than disenfectant: that is, it prevents the formation rather than changes the nature of putrid gases. The advantages of carbolic acid in surgery for cleansing dressings, sponges, bedding, floors, etc., is very great; and the cost need be but slight, since the impurity of the coal-tar creasote used is a matter of no consequence, the alcohols being alone soluble. A convenient way of keeping it for hospital use is as follows: Take a barrel to which a spigot is fitted; fill it half full of water, warm by preference; put into this two pints of the impure acid, and shake the mixture thoroughly; then fill the barrel with water, and set it up on its end; some of the impurities will rise to the top and some settle to the bottom. The liquid can be drawn off as required and diluted for use; a teacupful of it in a half gallon of water is sufficiently strong for scrubbing floors, etc. Bedding can be thoroughly disenfected by simple immersion in it."

On the above subject, we take a few remarks from a lecture by Dr. Crace Calvert, published in the Journal of the Society of Arts, France. The drawback to the use of carbolic acid, has been its tarry and sulphurous odor. Dr. Calvert has discovered a process by which the acid is deprived of all disagreeable odor and tarry flavor. It is distinguished from that of Laurent, in being soluble in twenty parts of water, whereas the later requires thirty-three. The antiseptic properties of carbolic acid are so powerful, that even $\frac{1}{5000}$th will prevent the fermentation or putrefaction for months, of blood, urine, paste, fæces, ect.; in fact, its vapor alone is sufficient to preserve meat in confined places for weeks; and $\frac{1}{1000}$th has been found sufficient to keep sewage sweet. Among the articles to which this acid has given rise, Dr. Calvert mentions *picric acid*, which is produced by the action of nitric acid, forming, with the carbolic, a trinitrophenic acid. Aside from its value in the arts, picric acid is an efficacious remedy in intermittent fevers. Persons affected with such types of fever upon whom quinine has lost all its beneficial effects, derive wonderful benefits from the use of this acid and picrates, as Dr. Ashland has proved at the Military Hospital at Dunkinfield Its cheapness and the fact that it is not dangerous, and is borne well by the stomach, adds to its value.

Dr. HOLMES COOTE (*British Medical Journal*, April 25th), speaks in high terms of the use of pure carbolic acid in primary syphilitic sores; reporting its successful use in twelve cases of soft chancre, effecting a cure in from ten to fourteen days.

The Boston correspondent, "G.," of the *Medical and Surgical Reporter* (February 15th), gives the following formula, used successfully as a lotion in diphtheria, putrid sore throat, sloughing, and phagedenic ulcers.

| | | |
|---|---|---|
| *R.* Acidi Carbolici - - - - - - - | gtt xxv. | |
| " Acetici, - - - - - - - | f drachm ss. | |
| Glycerine, - - - - - - - | f drachm ij. | |
| Aquæ destil, - - - - - - - | f oz. vj. | M. |

